# Artificial intelligence in gynecology and obstetrics: from the enthusiasm of use in practice to the challenges of implementation

**DOI:** 10.61622/rbgo/2024rbgo41

**Published:** 2024-05-27

**Authors:** Yago Tavares Pinheiro, Richardson Augusto Rosendo da Silva

**Affiliations:** 1 Universidade Federal do Rio Grande do Norte Natal RN Brazil Universidade Federal do Rio Grande do Norte, Natal, RN, Brazil.

## Dear Editor,

**Figure 1:**
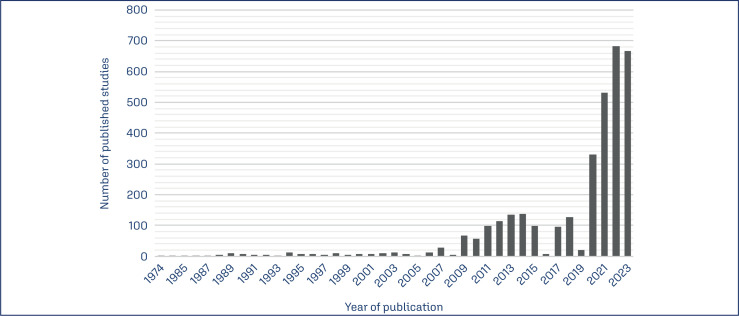
Number of studies published annually on artificial intelligence or machine learning around Gynecology and Obstetrics according to the MEDLINE database

Artificial intelligence (AI) and Machine Learning (ML) have been the subject of discussion among many professionals, researchers, and managers working in the fields of gynecology and obstetrics.^(1,2)^ In a simple search in the MEDLINE database with the descriptors "artificial intelligence" OR "machine learning", we identified 278,723 studies published until December 2023 on the topic. By delimiting the searches to the area of gynecology and obstetrics, in the same database, from the application of the search strategy ((machine learning) OR (artificial intelligence) AND (Obstetrics OR Gynecology)) it was possible to identify 3,359 studies published until December 2023. We noted that the number of studies related to the topic published in the last five years (2018 to 2023) increased almost four times compared to the previous five-year period (2012 to 2017) (Figure 1).

Given the information presented above, the growing interest in investigating the application of AI and ML in the fields of Gynecology and Obstetrics becomes evident. This interest possibly arises from the potential benefits of using these methods in clinical practice, among which we can mention: the possibility of analyzing structured data (imaging exams, genetic and electrophysiological tests, etc.); reduction of costs, and diagnostic and therapeutic errors inherent to practical human activity; and extracting information from a large population to make inferences about disease risk or predicting outcomes.^(3,4)^ However, given the enthusiasm surrounding the use of an emerging method with considerable potential in clinical practice, it is important to note that its implementation depends on overcoming some challenges involving the availability, accuracy, integrity, and security of health data. However, it is observed that the findings in the scientific literature on the barriers and facilitators regarding the implementation of AI and ML in the areas of Gynecology and Obstetrics are still very discreet. Furthermore, the discussion between researchers, clinicians, and health managers is still very early. Therefore, despite the growing trend of scientific investigation into these resources in recent years, there is still an urgent need to intensify the evidence-based dialogue about the effects and implementation of AI and ML in practice.

Therefore, professionals, researchers, and managers must be encouraged to develop a task force to expand knowledge about AI and ML applied to gynecological and obstetric care, in addition to encouraging them to discuss global and local development challenges. and implementation of AI, the responsibility, guarantee, and security of recording health data, as well as the cost-effectiveness and ethical aspects related to the use of these tools.

## References

[1] Chomutare T, Tejedor M, Svenning TO, Marco-Ruiz L, Tayefi M, Lind K (2022). Artificial intelligence implementation in healthcare: a theory-based scoping review of Barriers and facilitators. Int J Environ Res Public Health.

[2] Dhombres F, Bonnard J, Bailly K, Maurice P, Papageorghiou AT, Jouannic JM (2022). Contributions of artificial intelligence reported in obstetrics and gynecology journals: systematic review. J Med Internet Res.

[3] Stanfill MH, Marc DT (2019). Health information management: implications of artificial intelligence on healthcare data and information management. Yearb Med Inform.

[4] Jiang F, Jiang Y, Zhi H, Dong Y, Li H, Ma S (2017). Artificial intelligence in healthcare: past, present and future. Stroke Vasc Neurol.

